# Brazilian Protocol for Sexually Transmitted Infections 2020: human T-cell lymphotropic virus (HTLV) infection

**DOI:** 10.1590/0037-8682-605-2020

**Published:** 2021-05-17

**Authors:** Carolina Rosadas, Carlos Brites, Denise Arakaki-Sanchez, Jorge Casseb, Ricardo Ishak

**Affiliations:** 1 Imperial College London, Department of Infectious Disease, London, United Kingdom.; 2 Universidade Federal da Bahia, Faculdade de Medicina, Salvador, BA, Brasil.; 3 Ministério da Saúde, Secretaria de Vigilância em Saúde, Brasília, DF, Brasil.; 4 Universidade de São Paulo, Faculdade de Medicina, São Paulo, SP, Brasil.; 5 Universidade Federal do Pará, Instituto de Ciências Biológicas, Belém, PA, Brasil.

**Keywords:** Human T-Cell lymphotropic virus 1, Sexually transmitted diseases, Diagnosis, Signs and symptoms, Disease prevention

## Abstract

This article addresses the Human T-lymphotropic virus (HTLV). This subject comprises the Clinical Protocol and Therapeutic Guidelines for Comprehensive Care for People with Sexually Transmitted Infections, published by the Brazilian Ministry of Health. HTLV-1/2 infection is a public health problem globally, and Brazil has the largest number of individuals living with the virus. HTLV-1 causes several clinical manifestations of neoplasm (adult T-cell leukemia/lymphoma) and inflammatory nature, such as HTLV-1-associated myelopathy and other manifestations such as uveitis, arthritis, and infective dermatitis. These pathologies have high morbidity and mortality and negatively impact the quality of life of infected individuals. This review includes relevant information for health authorities professionals regarding viral transmission, diagnosis, treatment, and monitoring of individuals living with HTLV-1 and 2 in Brazil.

## Highlighted excerpt

HTLV-1/2 transmission can occur through blood transfusion and derivatives, injectable drug use, organ transplantation, unprotected sexual intercourse, and vertical transmission.

## FOREWORD

This article addresses Human T-lymphotropic virus (HTLV) infection. This subject comprises the Clinical Protocol and Therapeutic Guidelines (PCDT) for Comprehensive Care for People with Sexually Transmitted Infections (STI), published by the Health Surveillance Department of the Brazilian Ministry of Health. To elaborate the PCDT, selection and analysis of the evidence available in the literature were performed, and a panel of specialists discussed it. The document was approved by the National Committee for the Incorporation of Technologies in the Brazilian National Health System (Conitec)^1^ and updated by the team of specialists in STI in 2020^2^.

## EPIDEMIOLOGICAL ASPECTS

HTLV-1 was described in patients with adult T-cell leukemia/lymphoma and, like HTLV-2^3^ ^-^ ^6^, classified in the *Retroviridae* family, genus *Deltaretrovirus* ^7^. There are six molecular subtypes (a, b, c, d, e, f) of HTLV-1^8^ ^-^ ^10^and four (a, b, c, d) of HTLV-2^11^ ^-^ ^14^; and two other types, HTLV-3 and HTLV-4, which have been described in isolated areas of forests in Cameroon, a country in the western region of Central Africa, and not yet associated with clinical manifestations^15^ ^-^ ^17^.

HTLV-1/2 infection results from the transmission of infected lymphocytes, present in body fluids (blood, semen, vaginal secretion, and mother's milk), by transfusion of blood and derivatives, intravenous drug use, organ transplantation, unprotected sexual intercourse, and vertical transmission. Vertical transmission can occur by the placental route, during birth, and mainly by breastfeeding^18^ ^-^ ^25^. HTLV-1 proviral load and exposure time are related to the increased risk of transmission, especially during sexual intercourse or breastfeeding^26^. The risk associated with the transfusion of blood and its derivatives was significantly reduced, with the introduction of systematic screening of blood and organs and blood components' leukoreduction^27^ ^,^ ^28^.

Sexual contact is an important route of HTLV-1 and HTLV-2 dissemination in urban, rural, and indigenous areas^12^ ^,^ ^29^ ^,^ ^30^. In urban areas, infection is most common among women^31^ ^-^ ^33^. However, among indigenous communities, the transmission effectiveness shows no difference between the sexes^12^ ^,^ ^29^ ^,^ ^34^. Sexual transmission is associated with unprotected sex practices, sexual partnership with intravenous drug users, and the presence of other STI^35^ ^-^ ^37^.

HTLV-1 and HTLV-2 are distributed worldwide^18^. Brazil has variable frequencies, ranging from 0.01 to 1.35% in the general population^28^ ^,^ ^38^ ^,^ ^39^, according to the geographical area and behavioral risk factors^12^ ^,^ ^18^ ^,^ ^40^ ^,^ ^41^. Groups with higher vulnerability to infection by both viruses include (i) intravenous drug users, (ii) sex workers, (iii) men who have sex with men, (iv) individuals submitted to blood transfusion before 1993, and (v) sexual partners of individuals with known HTLV infection. The decrease in HTLV-1 prevalence among blood donors throughout the years^28^ ^,^ ^38^is a privileged situation in Brazil, promoted since 1993^42^ with the mandatory screening regulation of blood and its products.

The seroepidemiological studies for HTLV-1/2 are based on the detection of specific antibodies. It is important to emphasize that few population studies were conducted adequately. Therefore, a significant part of the epidemiological information about HTLV-1/2 derived from old studies, which often do not sufficiently define incidence and prevalence rates, shows conflicting results and does not allow the definition of precise prevention and control measures^18^ ^,^ ^39^.

HTLV-2, considered an ancestral infection, is apparently well adapted to humans, with rare clinical manifestations^5^ ^,^ ^43^ ^-^ ^48^. HTLV-2 is usually used as a marker of human migrations after the departure from the African continent^49^ ^,^ ^50^.

## CLINICAL ASPECTS

Retroviruses integrate with the nucleic acid in the infected cell and establish a viral persistence, leading to the virus maintenance and the different outcomes of the infection. HTLV-1 is associated with an aggressive malignant disease, adult T-cell leukemia/lymphoma (ATL)^51^ ^,^ ^52^, and the neurodegenerative disease HTLV-1 associated myelopathy (HAM)^53^ ^-^ ^57^.

HTLV-1 infection shows a great variety of interactions with the human host and important clinical manifestations have been recognized in the eye^58^ ^-^ ^61^, skin^61^ ^,^ ^62^, lung^61^ ^,^ ^63^ ^-^ ^65^, joints^66^ ^-^ ^68^, thyroid^69^ ^,^ ^70^, heart^61^ ^,^ ^71^ ^,^ ^72^, intestines^61^ ^,^ ^73^and bladder^61^ ^,^ ^74^ ^,^ ^75^, among others. The broad spectrum of diseases reveals the infection's clinical complexity, which requires multidisciplinary attention for the infected patients' care. Although the clinical outcome of the HTLV-1 infections is considered low (5%), the number of clinical cases associated with HTLV-1 infection can reach a higher level and still needs to be better defined^55^. Intermediate clinical manifestations can be frequent before HAM occurs^76^ ^,^ ^77^. The proviral load in HTLV-1 infection is important in disease progression^78^ ^,^ ^79^, and is usually lower in asymptomatic individuals compared with those who present HTLV-1 associated diseases.

## HTLV-1 ASSOCIATED MYELOPATHY

HAM occurs in about 4% of HTLV carriers, although clinical manifestations may affect more than 10% of them^77^. HAM manifests predominantly in the fourth and fifth decades of life, being uncommon before 20 or after 70 years of age. Generally, it starts insidiously and progresses slowly, especially among women: HAM cases in women are two to three times higher than that observed among men. Gait disturbances are a consequence to the gradual decrease in muscle strength and spasticity of the lower limbs^80^, leading to the need, over time, for walking aids (with the support of canes and walkers) and may evolve into the use of a wheelchair. The time of evolution varies, from months to decades. The symptoms of vesicointestinal and sexual dysfunction can be the initial complaints of the affected individual. Generally, HAM is characterized by urinary urge incontinence, intestinal constipation, and erectile dysfunction in the male population. The neurological clinical picture may be associated with multisystemic processes such as dermatitis, uveitis, pneumonia, besides cognitive alterations^81^ ^,^ ^82^. The diagnosis of HAM is rather critical since its early treatment may lead to a more effective therapeutic response^83^ and better prognosis when instituted up to five years after the first symptoms.

Proviral load levels correlate with the progression of the disease, especially with muscle weakness. Although the magnitude of the proviral load in peripheral blood is associated with HAM, it is not the sole diagnostic or prognostic factor of the pathology^84^. Proviral load in cerebrospinal fluid is important to define the progression of HAM since HTLV-1 infected cells in the central nervous system accelerate the local inflammatory process^26^ ^,^ ^85^ ^-^ ^87^. However, other prognostic value markers should be evaluated to identify people at higher risk of illness^88^ ^-^ ^90^. 

## ADULT T-CELL LEUKEMIA/LYMPHOMA

The neoplasm of peripheral T-cells caused by HTLV-1 presents itself with leukocytosis, characterized by the presence of abnormal lymphocytes (flower cells) and, clinically, by lymphadenopathies, skin lesions, dysfunction of multiple organs resulting from the invasion of the neoplastic cells, in addition to the presence of opportunistic infections. Elevated levels of the enzyme lactate dehydrogenase and hypercalcemia are characteristic. In Japan, there are over one million carriers and the incidence of ATL varies from 0.6 to 0.7 per 1000 persons/year^91^. The risk of illness is higher in men, and symptoms begin 20 to 30 years after infection^92^. Rarely, ATL occurs before 30 years of age; however, its frequency tends to increase to reach those with 70 years of age. In Japan, where the probability of developing ATL is 5%, risk factors are: (i) maternal transmission, (ii) older age, (iii) increased proviral load in peripheral blood, (iv) family history of ATL, and (v) prior positive testing for anti-HTLV-1^93^ ^,^ ^94^. ATL is rare in other countries, not reaching 2% of cases^95^, despite evidence of lack of diagnosis^96^ ^,^ ^97^. 

**FIGURE 1:**
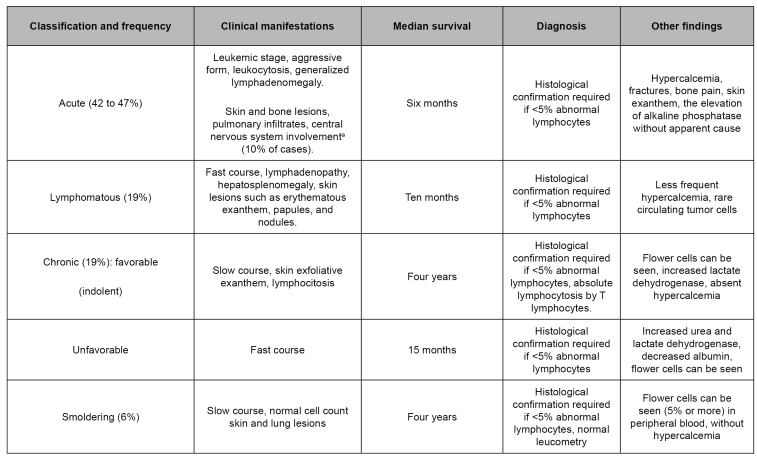
Classification and characteristics of adult T-cell leukemia/lymphoma.

**Source:** adapted from Nosaka et al. 2017^93^; Iwanaga et al. 2010^94^; Phillips et al. 2010^95^; Rosadas et al. 2020^96^.

**Note:** a) Need for intrathecal chemotherapy.

**FIGURE 2:**
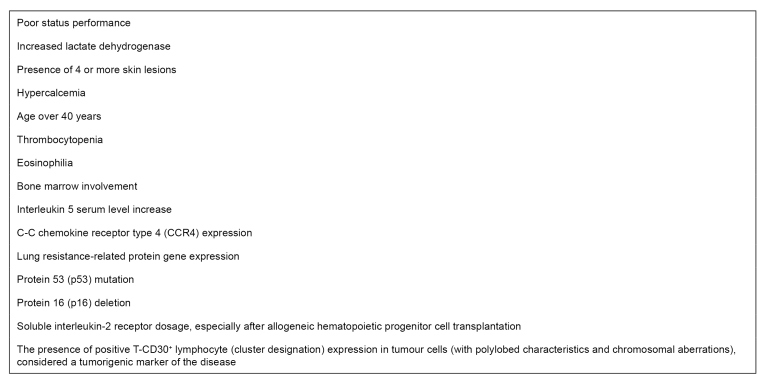
Adult T-cell leukemia/lymphoma worst prognosis predictors.

**Source:** adapted from Iwanaga et al. 2010^94^.

Four clinical forms of ATL are recognized^98^, which take into account the presence and severity of the leukemic manifestations, in addition to altered laboratory tests, such as increased lactate dehydrogenase and hypercalcemia. This classification is described in Figure 1, and the factors that predict worse prognosis, including those mentioned above, are found in Figure 2 ^51^ ^,^ ^98^ ^-^ ^101^.

## DERMATOLOGICAL ALTERATIONS IN INDIVIDUALS WITH HTLV

In addition to the clinical manifestations classically associated with HTLV-1 in the skin, such as infective dermatitis and the cutaneous manifestations of ATL, other dermatological affections attributed to the infection have been described as serious forms of scabies (especially in HIV-1 coinfected individuals)^102^, ichthyoses, seborrheic dermatitis, and dermatophytoses^103^.

At first, infective dermatitis was described in Jamaican children infected by HTLV-1^104^, mainly when vertical transmission occurs, although the disease can also affect adolescents and adults^105^. Infective dermatitis is characterized by erythematous-desquamative lesions, which generally involve the scalp, retro auricular regions, neck, face, armpits, and inguinal region. Typically, it is associated with infection by Gram-positive bacteria such as *Streptococcus beta-hemoliticus* and *Staphylococcus aureus*. According to a case series study, almost half of the individuals who had long-term follow-up were also diagnosed with HAM^106^. The differential diagnosis includes other causes of chronic eczemas, such as atopic dermatitis and seborrheic dermatitis^106^. Presence of the characteristic lesions, chronic rhinorrhea, recurrent chronic dermatitis, and positive serology for HTLV are the main criteria for diagnosing infective dermatitis, whose treatment consists of administering antibiotics with topical use of corticosteroids, combined or not with antifungals.

Dermatological alterations in ATL vary in presentation (erythroderma, papules, nodules, infiltrating lesions, or erythematous plaques) and depend on the disease stage; nodulations are more frequent in severe forms, especially in the acute, lymphomatous, or cutaneous primary tumoral form^107^. The lesions may evolve indolently and modify with the use of corticosteroids. Histopathological evaluation is essential for specific diagnosis.

## UVEITIS IN INDIVIDUALS WITH HTLV-1

In Japan, uveitis was first reported in 1989^108^. Most common in people in age up to 50 years and a little more frequent in women, its exact incidence among HTLV-1 carriers remains uncertain. The disease is manifested by visual disorders, including 'floaters' and blurred or hazy vision, and it is bilateral in almost half of the affected people^109^. Eye signs include iritis, vitreous opacities, retinal vasculitis, and retinal hemorrhages and exudates. There is a good patient response to topical or systemic corticosteroids, although recurrence is common with therapy discontinuation.

## COINFECTIONS IN INDIVIDUALS WITH HTLV

HTLV-infected individuals may present some coinfections, more frequently than the general population, either by sharing infection routes or as a consequence of the immunological alterations induced by the infection itself. Moreover, HTLV can alter the natural course of some coinfections.

In HIV coinfection, for example, the evidence suggests a neutral or even protective role for those coinfected by HTLV-2^110^. However, if the coinfection is HIV-1/HTLV-1, the existing data show a higher risk of death, both in adults and in children^111^. The reasons for these findings are not very clear. A hypothesis for the lack of clinical benefit is the delay in introducing the antiretroviral therapy due to the increase in the T-CD4^+^ cells count caused by HTLV-1. Coinfected individuals treated with antiretroviral therapy and with HIV-1 viral suppression present similar survival time to those monoinfected under the same conditions; however, in those with a detectable viral load, the survival of coinfected individuals is significantly lower^112^.

Regarding coinfection with hepatitis C virus (HCV), existing data are conflicting: while some studies show an increase in HCV viremia and a lower probability of spontaneous clearance of the infection^113^, others suggest a higher chance of elimination of this virus in HIV-1 and HTLV-coinfected individuals, probably due to the immunomodulation caused by HTLV in this group of individuals, resulting from the high production of proinflammatory cytokines^114^. Moreover, studies are suggesting less hepatic damage in triple infected individuals - with HIV, HTLV, and HCV- and a greater chance of spontaneous clearance of HCV^115^ ^,^ ^116^.

Individuals with HTLV-1 and *Strongyloides stercoralis* coinfection suffer a negative impact in the course of both infections, becoming more susceptible to more severe forms of strongyloidiasis, therapeutic resistance, in addition to presenting a higher HTLV-1 proviral load and a higher risk of HTLV-1 vertical transmission^117^ ^-^ ^126^.

Individuals with HTLV-1 present a higher risk of infection by *Mycobacterium tuberculosis* ^127^ ^-^ ^132^, but the clinical impact is not clear.

## DIAGNOSIS

**FIGURE 3:**
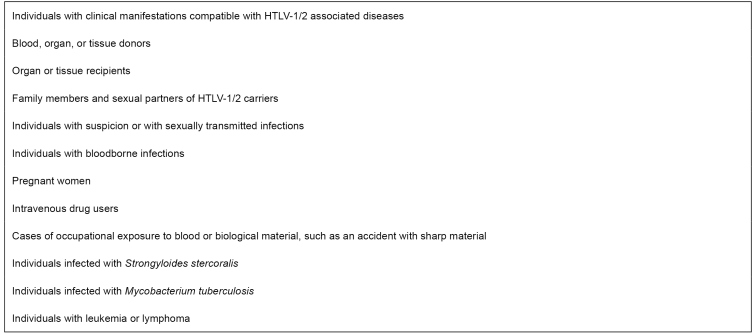
Indications for laboratory testing for the human T-cell lymphotropic virus (HTLV-1/2).

**FIGURE 4:**
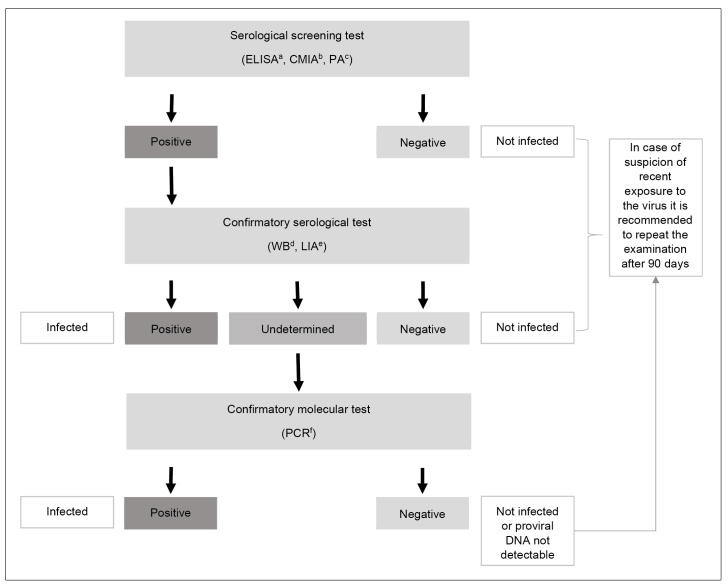
Recommendations for human T-cell lymphotropic virus (HTLV-1/2) infection laboratory diagnosis.

**Notes:** a) ELISA: immunoenzymatic assay; b) CMIA: chemiluminescence; c) PA: particle agglutination; d) WB: Western blot; e) LIA: line immunoassay; f) PCR: polymerase chain reaction.

In Brazil, routine testing for HTLV-1/2 in blood and organ donors has been performed since 1993 and 2009, respectively^42^ ^,^ ^133^. In both cases, the infection is a criterion for donor exclusion. Although there is no national policy for HTLV-1/2 antenatal screening in Brazil, the test is done as a routine in some states. The MS/SCTIE Portaria no. 23, of May 31, 2016, included the Western blot (Wb) test and the polymerase chain reaction (PCR) to confirm HTLV-1 infection among patients suspected of ATL assisted by the Brazilian National Health System (SUS)^134^. Figure 3 shows the indications for HTLV-1/2 testing. Laboratory diagnosis must be performed using screening tests, followed by confirmatory tests in a different blood sample when screening test results are positive^135^ ^-^ ^137^(Figure 4).

The screening tests are used for detecting antibodies against HTLV-1/2 in plasma or serum. The laboratory techniques for performing these tests include (i) immunoenzymatic reaction, (ii) chemiluminescence, and (iii) particle agglutination^136^. The screening tests present high sensitivity. The negative result excludes infection - unless there is evidence of recent exposure to the virus when it is recommended to repeat the test after 90 days^24^ ^,^ ^25^. The specificity of screening tests in Brazil varies from 92 to 99.5%. It is highly recommended to perform confirmatory tests to exclude false-positive results in the screening tests^136^ ^-^ ^138^.

The confirmatory tests identify antibodies against different HTLV-1 and HTLV-2 antigens or amplify and identify proviral genetic material, usually in peripheral blood lymphocytes. Confirmatory and viral typing tests are (i) Wb, (ii) line immunoassay (LIA), and (iii) PCR^136^.

Usually, Wb and LIA are sufficient for diagnosis; however, in some cases, undetermined or undefined results may occur regarding the type of HTLV^139^ ^-^ ^149^, more frequently in individuals infected by HTLV-2 or HIV-1 or both^141^ ^,^ ^150^. LIA presents greater accuracy in confirming HTLV-1 and HTLV-2 infection when compared to Wb^151^ ^,^ ^152^. Indeterminate or untyped results by Wb or LIA must be submitted to qualitative or quantitative PCR: nested PCR (nPCR) and real-time PCR (RT-PCR) are used. RT-PCR enables not only the quantification of the HTLV-1/2 proviral load but also the stratification of the risk of developing HTLV-1 associated diseases^26^ ^,^ ^93^ ^,^ ^94^ ^,^ ^142^ ^,^ ^153^ ^-^ ^155^. The detection of viral RNA is not used in the clinical routine, since viremia is low or absent, even in individuals with HAM^156^ ^,^ ^157^.

At the time of this publication, a molecular test for HTLV-1/2 is not commercially available. The tests used are in-house, requiring prior validation^155^ ^,^ ^158^ ^-^ ^161^. The absence of commercial tests and standardization of national protocols makes the implementation of molecular testing in the routine and the comparison of results obtained in different laboratories difficult^162^ ^,^ ^163^. Some individuals infected by HTLV-1/2 may present undetectable proviral load^164^ ^-^ ^166^. In these cases, it is possible to perform nPCR of higher sensitivity than RT-PCR. Another alternative is to perform a confirmatory serological test (if not yet performed) or to request consecutive samples for follow-up^148^. 

There is evidence that the duration of the immunological window period in HTLV-1/2 infection for antibody detection varies from 16 to 39 days after organ transplantation, and for the proviral genetic material, from 16 to 23 days after infection^167^. A study conducted with individuals infected by blood transfusion showed a median seroconversion of 51 days (36 to 72 days)^25^. It is important to emphasize that the methodologies available when this study was developed did not have the same sensitivity as the current diagnostic methods^168^.

## TREATMENT

The therapy for HTLV-1 infection consists of interventions directed to the complications resulting from the disease^169^ ^,^ ^170^. In 2016, Conitec^170^, and in 2019-2020, the International Retrovirology Association published recommendations for ATL and HAM treatment^171^ ^,^ ^172^. The use of zidovudine associated with interferon-alpha was authorized for the treatment of ATL by the publication of MS/SVS Portaria no. 54 on Jul 18, 2016^2^ ^,^ ^170^. The therapeutic regimens vary according to clinical presentation, progression of symptoms, and local availability of medications.

Infected people must be accompanied in the specialized service to receive psychological support, with particular attention to the early diagnosis of clinical manifestations associated with the infection.

## SURVEILLANCE, PREVENTION, AND CONTROL

Despite being described some decades ago, HTLV infection remains relatively unknown to the general population and health professionals. In the services that assist the infected individuals, the approach should focus not only on the aspects of the risk of becoming sick^173^ but also on preventing the transmission of infection. 

After a positive diagnosis for HTLV-1/2 infection, the sexual partners should be invited to undergo serological screening, and those with positive tests must be referred for counseling and appropriate follow-up. Such counseling should include information about the chronicity of the infection and the relevance of long-term clinical follow-up^169^ ^,^ ^174^. It is important to clarify the initial clinical manifestations and their progression, the transmission mechanisms, and their prevention. The donation of blood, semen, solid organs or tissues and breastfeeding are strongly discouraged. 

In HIV and other STI specialized clinical centres, it is important to include HTLV screening in the routine of care. HTLV-infected individual must be oriented about the risk of sexual transmission, serodiscordant sexual partners, and condom use - which may be interrupted during the fertile period when there is a firm decision to become pregnant and following medical counselling and recommendation^174^.

In Brazil, given the scarcity of material available for health professionals and the general population, several initiatives have been developed by academic groups and non-governmental organizations to disseminate information about HTLV-1/2. Among the organizations and initiatives with this purpose, the following should be highlighted: the Research Support Center on Retroviruses (NAP-Retroviruses) of the University of São Paulo; the Hemominas Foundation Journals on HTLV infection; the HTLVida Association; and the Vitamóre Group - Association of HTLV Carriers.

The lack of a national register system impairs the identification of the actual scenario of the infection in the country and, therefore, the implementation of specific public health policies. It is essential to highlight that case notification is one of the pillars of confrontation and research about HTLV-1 in countries like Japan, England, Spain, and Martinique island^175^ ^-^ ^178^.

## SPECIAL POPULATIONS

### Pregnant women

In Brazil, HTLV-1/2 prevalence in pregnant women can reach 1% in certain regions of the country (Table 1)^159^ ^,^ ^179^ ^-^ ^196^. Despite reports about the development of HTLV-associated diseases in pregnancy (HAM, ATL), there is no consistent evidence about the impact on the pregnancy-puerperium cycle^23^. However, childhood infection is associated with an increased risk of developing diseases associated with HTLV-1, especially ATL that has a high lethality^23^ ^,^ ^197^ ^,^ ^198^. Therefore, prevention of mother to child transmission is essential to reduce the incidence of diseases associated with the virus^23^ ^,^ ^96^ ^,^ ^137^. 

**TABLE 1:** Prevalence of HTLV-1/2 infection in pregnant women in different Brazilian states.

Region/State	Prevalence (%)	n	References^a^
**North**			
Pará	0.6	324	Guerra et al. 2018^188^ ^b^
	0.3	13,382	Sequeira et al. 2012^192^
Amazonas	0	674	Machado Filho et al. 2010^194^
**Northeast**			
Alagoas	0.2	54,813	Moura et al. 2015^179^
Bahia	0.14	692	Boa-Sorte et al. 2014^190^ ^c^
	1.05	2,766	Mello et al. 2014^191^
	0.98	408	Magalhães et al. 2008^195^
	0.84	6,754	Bittencourt et al. 2001^183^
	0.88	1,024	Santos et al. 1995^185^
Maranhão	0.7	713	Mendes et al. 2020^186^
	0.3	2,044	Guimarães de Souza et al. 2012^193^
Ceará	0.12	814	Broutet et al. 1996^184^
**Midwest**			
Mato Grosso do Sul	0.13	116,689	Dal Fabbro et al. 2008^196^
	0.1	32,512	Figueiró Filho et al. 2007^180^
Goiás	0.1	15,485	Oliveira et al. 2006^181^
**Southeast**			
Rio de Janeiro	0.74	1,628	Barmpas et al. 2019^187^
	0.66	1,204	Monteiro et al. 2014^189^
São Paulo	0.1	913	Olbrich Neto et al, 2004^182^
**South**			
Paraná	0.31	643	Medeiros et al. 2018^159^ ^d^

a) Only studies with confirmatory tests for HTLV-1/2 infection were included; b) Adolescent pregnant women; c) Study with blood samples on filter paper; d) High-risk pregnant women.

Since breastfeeding is the main mother to child transmission route of HTLV-1/2^135^ ^,^ ^199^ ^-^ ^204^and there is no vaccine against the infection or even any curative treatment, breastfeeding is contraindicated in mothers infected by the virus. For these women, the use of lactation inhibitors is recommended and the provision of infants with milk formula substitutes^2^. Universal antenatal HTLV-1/2 infection screening is not provided by the SUS, but it is recommended to test all pregnant women, followed by counselling for those infected and their relatives, allowing the effective implementation of prevention strategies. 

### Indigenous peoples

The vertical and sexual transmission routes are essential for HTLV maintenance in epidemiologically closed or semi-closed communities, as it occurs with HTLV-2c, which is prevalent among indigenous people residing in the Brazilian Amazon and urban areas^12^ ^,^ ^13^ ^,^ ^205^ ^-^ ^209^. It is worth remembering that intrafamiliar infection in the Kayapó communities is important and it is observed the transmission of the virus between two or three generations and in more than 20% of infected children under nine years old^12^. Vertical transmission maintains the virus in high endemicity since the usual nonbreastfeeding procedures by infected mothers are not followed regularly^205^. The increasing number of reports associating diseases with HTLV-2^5^ ^,^ ^43^ ^-^ ^48^infections requires special attention to the indigenous communities located in areas of high virus endemicity in the Brazilian Amazon^39^.

## CONCLUSIONS

Although HTLV infection is neglected, Brazil has produced several initiatives directed towards the prevention of HTLV-1 infection and disease. The complications with relevant clinical consequences, such as HTLV-1 associated myelopathy and T-cell leukemia/lymphoma, can be minimized with access to services offered by the SUS. The low complexity cases can be assisted at the health centers and, when necessary, forwarded to the specialized centers for treatment, rehabilitation, and social support. Despite the severe consequences that the infection can have on people's lives, its control still represents a public health challenge. National epidemiological studies, development and validation of diagnostic tests, and elaboration of clinical protocols with new therapeutic options can define public policies and specific actions towards the approach, prevention, control, and adequate treatment of HTLV-1/2 infection in Brazil.
